# Disseminated *Mycobacterium avium* infection in a cat on long-term ciclosporin therapy and potential latent infection of an in-contact cat

**DOI:** 10.1177/20551169221109442

**Published:** 2022-08-10

**Authors:** Jade Webster, Francesco Marchesi, Danièlle Gunn-Moore, Hayley Haining, Alison E Ridyard

**Affiliations:** 1 Small Animal Hospital, University of Glasgow, Glasgow, UK; 2 Veterinary Diagnostic Services, University of Glasgow, Glasgow, UK; 3 Royal (Dick) School of Veterinary Studies and The Roslin Institute, University of Edinburgh, Edinburgh, UK

**Keywords:** *Mycobacterium avium*, disseminated MAC, ciclosporin, latent infection, neurological

## Abstract

**Case summary:**

An 8-year-old domestic shorthair cat receiving long-term ciclosporin treatment was evaluated for a history of weight loss and hyporexia. The main clinical finding was a cluster of enlarged mesenteric lymph nodes. Cytological examination of fine-needle aspirates showed granulomatous inflammation with abundant acid-fast bacilli. A diagnosis of *Mycobacterium avium* complex (MAC) infection was confirmed by PCR. The cat’s clinical condition deteriorated rapidly despite appropriate antibiotic treatment and it was euthanased 2 weeks after initial presentation due to development of severe paraparesis and ataxia. Post-mortem examination revealed granulomatous inflammation affecting multiple lymph nodes and other organs with intrahistiocytic acid-fast bacilli consistent with mycobacteria when stained using Ziehl–Neelsen stain. Another cat in the same household was screened for infection using the interferon gamma release assay (IGRA), with the result being consistent with infection by non-tuberculous mycobacteria (NTM), which includes MAC; however, it had no grossly detectable disease.

**Relevance and novel information:**

This case report is an unusual presentation of disseminated MAC infection in a cat, which remains a rare diagnosis. Clinicians should be aware of unusual and rare presentations of this infection. The clinical findings, progression of disease and histopathology results add to the current clinical database for feline disseminated MAC infections. Another cat in the same household tested positive for NTM by IGRA without any gross disease. This was suggestive of latent MAC infection which, to our knowledge, has not been previously reported in an in-contact cat.

## Case description

An 8-year-old female neutered domestic shorthair cat presented to the Small Animal Hospital, University of Glasgow, UK for investigation of enlarged mesenteric lymph nodes that had been detected as an incidental finding on an abdominal ultrasound scan 6 weeks earlier.

The cat had experienced an acute kidney injury of unknown origin as a kitten from which it had recovered; consequently, it had routine serum biochemistry performed annually. At the most recent annual vaccination and assessment, mild azotaemia (when evaluated using the International Renal Interest Society [IRIS] guidelines of creatinine <140 µmol/l) had been detected (creatinine 158 µmol/l [reference interval (RI) 71–212] and urea 9.6 mmol/l [RI 5.7–12.9]). Subsequently, a focused ultrasound of the urogenital tract showed bilateral structural changes consistent with chronic kidney disease (CKD). Incidentally, a cluster of enlarged mesenteric lymph nodes was detected during the scan, with the largest node measuring approximately 2 cm. As the cat was clinically well, further investigation was not performed until 6 weeks later when the cat developed weight loss, hyporexia and mild lethargy.

The cat had been diagnosed with allergic dermatitis causing head and neck excoriation 6 years prior to presentation, which was being managed with long-term ciclosporin administration (Atopica; Elanco) at a dosage of 30 mg q24h (equivalent to ~7 mg/kg) given per os (PO). In addition, the cat had recently been started on telmisartan (Semintra; Boehringer Ingelheim) at a dosage of 1 mg/kg q24h PO for IRIS stage 2 CKD, substage borderline proteinuria (urine protein:creatinine ratio 0.3).

The cat was indoor-only, and was fed on a commercial cooked cat food and had never been fed raw meat or given unpasteurised milk. There was no known access to rodents and the only hunting history was between the ages of 4 months to 5 years old, when the cat had had access to a bird nest in the owner’s loft.

On physical examination, the cat was in good body condition (body condition score 4/9) and weighed 4.1 kg. A small-to-medium-sized mass was palpable in the mid-abdomen; otherwise, physical examination was unremarkable.

Haematology and serum biochemistry revealed several abnormalities, detailed in Table 1. Of note was the absence of the previous azotaemia. The cat was negative for feline immunodeficiency virus (FIV) and feline leukaemia virus (FeLV), and urine specific gravity was 1.044.

**Table 1:** Summary of haematological and biochemical findings

Test	Result	RI
WBCs (× 10^9^/l)	19.04	5.5–15.5
Neutrophils (× 10^9^/l)	17.517	2.5–12.5
Band neutrophils (× 10^9^/l)	0.19	0
Lymphocytes (× 10^9^/l)	0.381	1.5–7
Potassium (mmol/l)	5.3	2.6–5.2
Urea (mmol/l)	9.4	2.7–9.2
Creatinine (µmol/l)	84	91–180
AST (U/l)	61	0–30

RI = reference interval; WBCs = white blood cells; AST = aspartate aminotransferase

**Figure 1:**
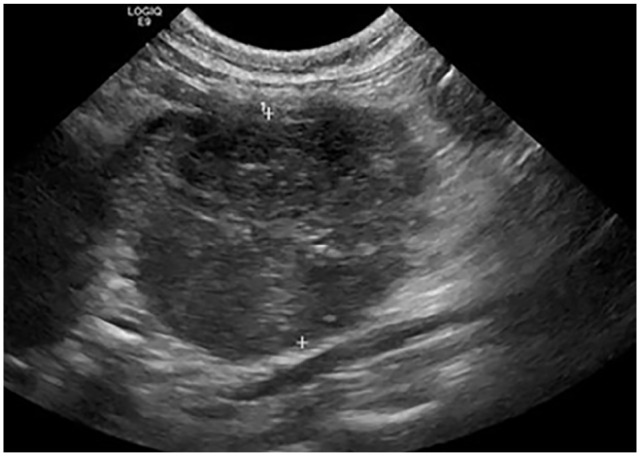
Abdominal ultrasound image of an enlarged mesenteric lymph node, measuring 2.77 cm in diameter

**Figure 2:**
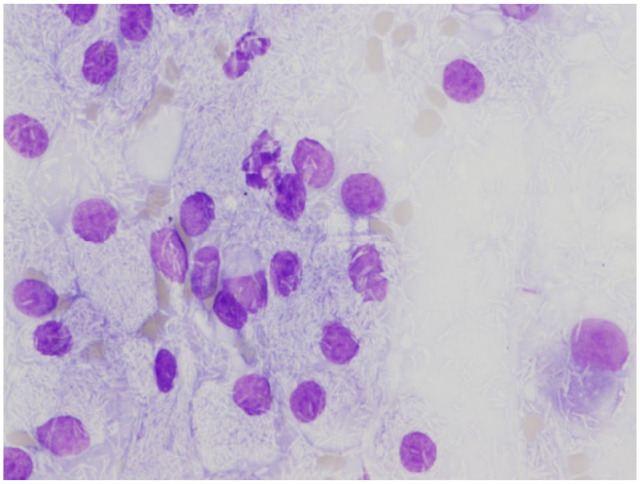
Cytology of abdominal lymph node aspirate showing multiple negatively staining rods present in the cytoplasm May–Grünwald Giemsa (× 600)

**Figure 3:**
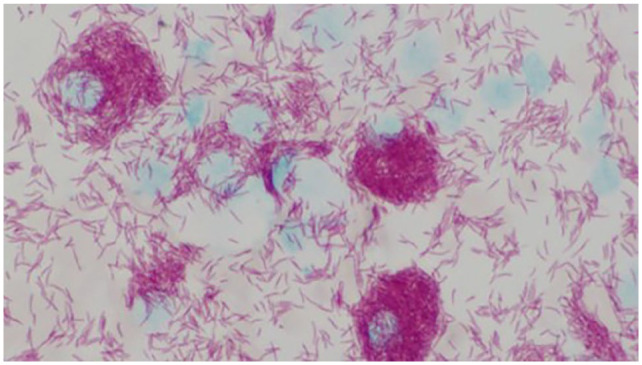
Cytology of the abdominal lymph node aspirates with Ziehl–Neelsen stain (× 600)

**Figure 4:**
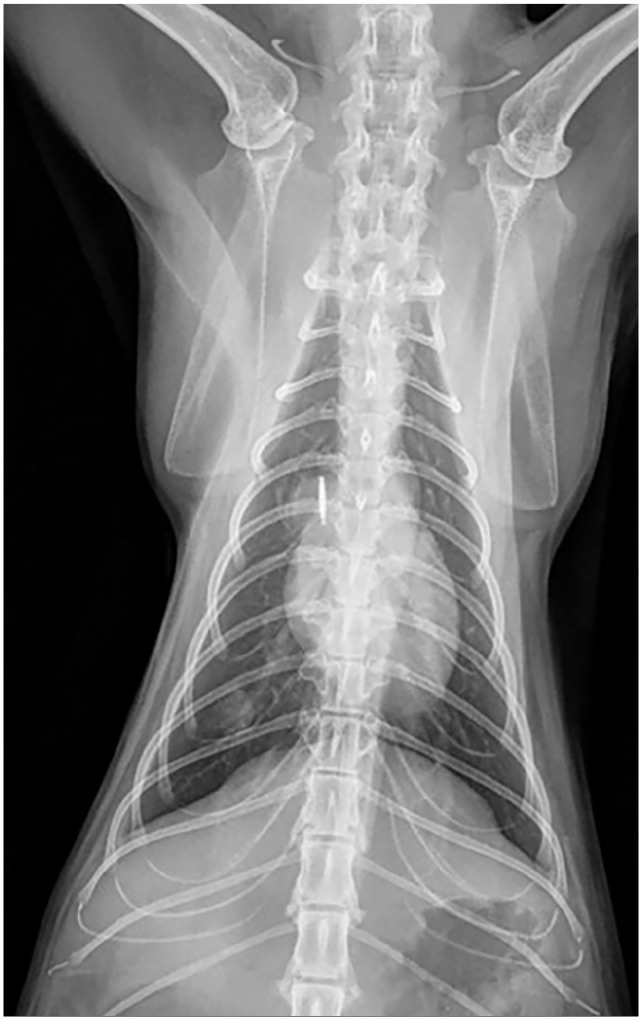
Dorsoventral thoracic radiograph showing a discrete soft tissue nodule in the right caudal lung lobe visible between the 9th and 10th ribs; this nodule is not, however, clearly identifiable on the lateral views

**Figure 5:**
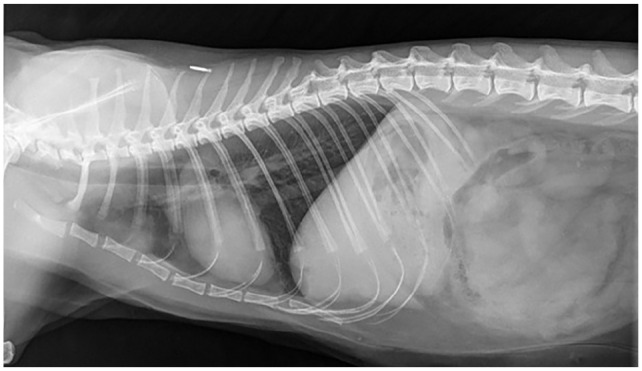
Left lateral thoracic radiograph showing sternal and tracheobronchial lymphadenomegaly

**Figure 6:**
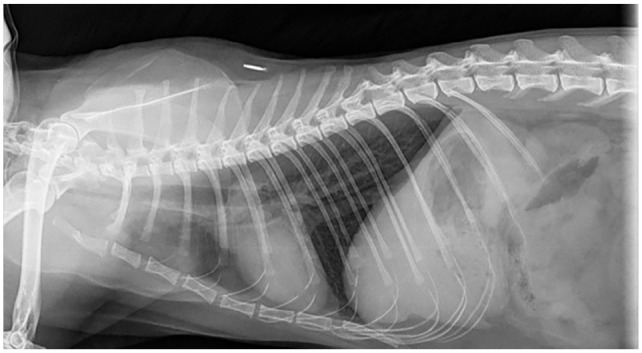
Right lateral thoracic radiograph showing sternal and tracheobronchial lymphadenomegaly

Abdominal ultrasound examination confirmed enlargement of the mesenteric lymph nodes (Figure 1), measuring almost 3 cm. Aspirates yielded a specimen containing large numbers of macrophages with intracellular non-staining rods when stained with May–Grünwald Giemsa (Figure 2). Ziehl–Neelsen staining showed large numbers of acid-fast bacilli with morphology consistent with mycobacterial infection (Figure 3). Thoracic radiographs (Figures 4–6) showed a bronchial pattern and sternal and tracheobronchial lymphadenomegaly.

At the follow-up assessment 5 days later, the abdominal masses were subjectively larger on palpation and the cat had lost >10% of its body weight in that time (weight now 3.65 kg). Fine-needle aspirates from the mesenteric lymph nodes were submitted for mycobacterial PCR to the Department of Microbiology, Leeds Teaching Hospital. Antibiotic therapy was initiated with azithromycin (Zithromax; Pfizer) at a dosage of 15 mg/kg q24h PO and pradofloxacin (Veraflox; Bayer) at a dosage of 5 mg/kg q24h PO, pending results. Maropitant (Cerenia; Zoetis) at a dosage of 1 mg/kg q24h PO was dispensed to address azithromycin-related nausea. Ciclosporin therapy was continued owing to the potential welfare implications of stopping treatment. Telmisartan treatment was stopped as it was deemed less necessary at this time. An oesophagostomy tube was placed under general anaesthesia to facilitate patient management and to ensure better owner compliance with antibiotic administration.

Four days after treatment was initiated, the cat was reportedly more hyporexic and lethargic. Azithromycin-related nausea was suspected and the dosage was reduced (12 mg/kg q24h), maropitant was continued and mirtazapine (0.5 mg/kg q48h PO) was prescribed. Supplementary feeding was provided via the oesophagostomy tube.

The cat re-presented 9 days after starting initial antibiotic therapy with progressive and severe ataxia and paraparesis. Neurological examination revealed severe ambulatory paraparesis. Cranial nerve examination was normal and there was no obvious neck or spinal pain. Thoracic limb withdrawal reflexes were poor/weak and pelvic limb proprioception was markedly decreased. Neurolocalisation was to either the cervical spine or representative of a multifocal process. Considering the guarded-to-poor prognosis, the cat was euthanased.

**Figure 7:**
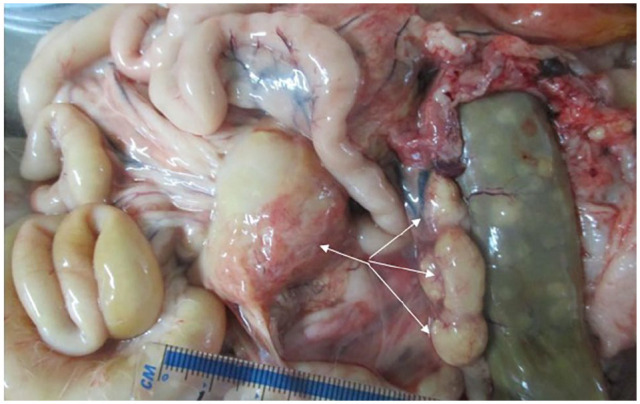
Abdominal cavity at post-mortem examination: massive enlargement of the mesenteric lymph nodes (arrows)

**Figure 8:**
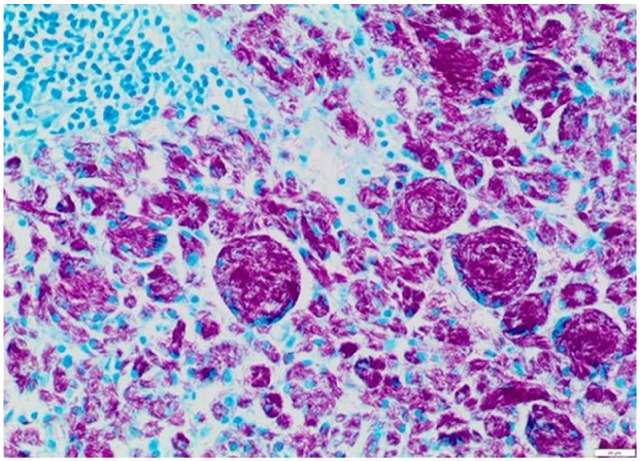
Histology of one of the mesenteric lymph nodes. Dense aggregates of macrophages and multinucleate cells with large numbers of intracytoplasmic acid-fast bacilli. Ziehl–Neelsen (× 200); scale bar = 20 µm

**Figure 9:**
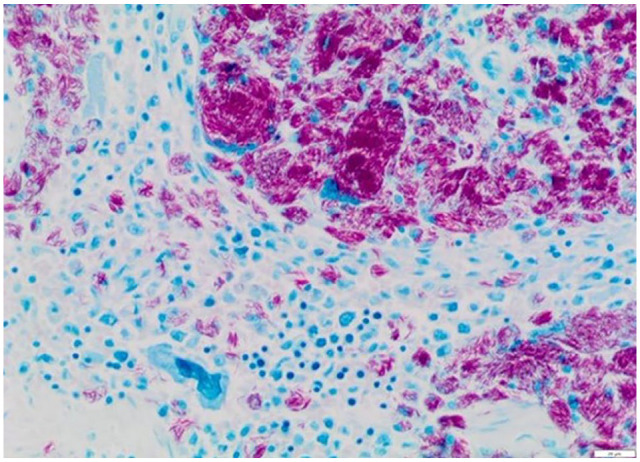
Histology of the spleen. Aggregates of macrophages and multinucleate cells with large numbers of intracytoplasmic acid-fast bacilli. Ziehl–Neelsen stain (× 200); scale bar = 20 µm

**Figure 10:**
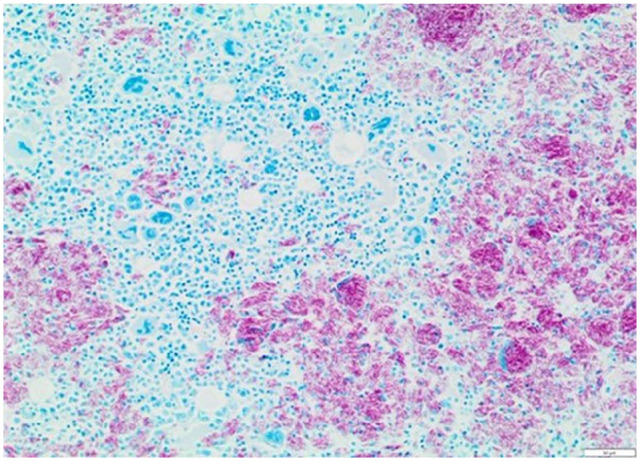
Histology of bone marrow. Aggregates of macrophages and multinucleate cells engulfing acid-fast bacilli admixed with residual portions of marrow with multilineage haematopoiesis. Ziehl–Neelsen (× 100); scale bar = 50 µm

Post-mortem examination revealed moderate-to-massive lymphadenomegaly with granulomatous inflammation affecting multiple lymph nodes: retropharyngeal, mediastinal, peribronchial, perisplenic, pancreatic and mesenteric lymph nodes, with the largest mesenteric lymph node measuring 70 × 50 × 30 mm (Figure 7). The tonsils were also enlarged. Histologically, many lymph nodes had >90% of the tissue effaced by sheets of epithelioid macrophages and multinucleate cells engulfing negatively stained bacilli. Granulomatous inflammation was also detected in lungs, liver, spleen, pancreas, intestine/colon, skin, diaphragm and bone marrow, with intrahistiocytic acid-fast bacilli (with morphology consistent with mycobacteria) confirmed by Ziehl–Neelson stain in multiple tissues (Figures 8–10). Focal involvement of the kidneys was also observed. The right caudal lung lobe had a focally extensive area of complete obliteration of the parenchyma and the medium-sized bronchus, shown by epithelioid macrophages containing acid-fast bacilli. The bone marrow had approximately 60–80% of the marrow tissue effaced by extensive and coalescing infiltrates of epithelioid macrophages and moderate numbers of Langhan-type multinucleate cells engulfing acid-fast bacilli.

Pathology findings provided no clear explanation for the paraparesis. Rare macrophages with intracytoplasmic acid-fast bacilli were found in the brainstem, cerebellum and pons, and the cervical and lumbar spinal cord (in the subdural space and adjacent to nerve roots). The brain/cerebellum and spinal cord had no obvious parenchymal granulomatous inflammation, and no granulomatous inflammation or acid-fast bacilli were found in other neural tissues examined.

At this time the results of the pre-mortem mycobacterium PCR confirmed *Mycobacterium avium* infection from the aspirates taken from the mesenteric lymph nodes.

The other, unrelated, cat in the same household, a 9-year-old male neutered domestic shorthair (also indoor-only and fed the same diet as the other cat) was assessed for infection. No lesions were detected on thoracic or abdominal imaging. Blood was sent for interferon gamma release assay (IGRA) assessment (Biobest Laboratories), the result of which was suggestive of infection with non-tuberculous mycobacteria (NTM). Considering the absence of clinical signs in this case, this likely represented a latent infection and regular monitoring was recommended.

## Discussion

Mycobacteria are a group of pathogens that can be broadly classified into two major categories: *Mycobacterium tuberculosis* complex (MTBC) and NTM. NTMs are ubiquitous in the environment, particularly in biotopes such as soil and water.^1^ *Mycobacterium avium* complex (MAC) is a group of slow-growing mycobacteria that cause opportunistic infections in the host and are often (erroneously) grouped with the MTBC pathogens due to indistinguishable clinical disease.^2^ MAC infection is the most frequently confirmed NTM infection in companion animals and is the third most common mycobacteria detected in cats in the UK, following *Mycobacterium microti* and *Mycobacterium bovis*.^3^

Feline mycobacterium infections (including *M avium* infections) most often result in cutaneous lesions, with disseminated disease occurring less commonly.^3^ The MAC group of organisms are the most likely of all of the NTM to produce disseminated disease in the cat, of which there here have been several reports.^4–19^ Abyssinian and Siamese breeds appear to be over-represented.^4,6,8,9^

Multiorgan involvement, as seen in this case, is a key feature of disseminated MAC infection, with haematogenous spread being the proposed mechanism of dissemination.^11^ Table 2^4–7,10–16,18,19^ and Table 3^4–19^ summarise the organ involvement and clinical manifestations of the previously reported cases of disseminated MAC. However, in contrast to many of these cases, and given the extent of the changes found on this cat’s post-mortem examination, there was more limited clinical evidence to suggest widespread dissemination in this case. The cat presented here was in good body condition, normothermic and had no peripheral lymphadenomegaly, there were no haematological changes suggestive of extensive bone marrow involvement and the pulmonary changes visible on radiography were restricted to an isolated pulmonary nodule that had been interpreted as atelectasis.

**Table 2:** Organs with confirmed involvement in previous disseminated *Mycobacterium avium* cases^4–7,10–16,18,19^

Organs involved	Cases (n = 24)
Lymph nodes	22 (92)
Lungs	18 (75)
Liver	15 (63)
Spleen	12 (50)
Bone marrow	11 (46)
Intestines	10 (42)
Kidney	5 (21)
Omentum	4 (17)
Brain	3 (13)
Mesentery	1 (4)
Peritoneum	1 (4)
Vulva	1 (4)
Pancreas	1 (4)
Skin	1 (4)

Data are n (%)

**Table 3:** Most commonly reported clinical manifestations in previous cases of disseminated *Mycobacterium avium* cases^4–19^

Clinical manifestation	Cases (n = 29)
Weight loss/poor BCS	23 (79)
Anorexia/hyporexia	15 (52)
Respiratory signs	14 (48)
Peripheral lymphadenomegaly	12 (41)
Pyrexia	12 (41)
Abdominal mass	7 (24)
Lethargy	7 (24)
Vomiting	5 (17)
Abdominal organomegaly	4 (14)
Neurological signs	3 (10)

Data are n (%)

BCS = body condition score

While disseminated disease is a feature of MAC in cats, there are only rare reports of cats with neurological manifestations. A solitary intracranial mass caused by MAC infection in the absence of disseminated disease or an identifiable primary lesion has been reported previously, and pyogranulomatous meningoencephalitis and extensive cerebral infarctions with acid-fast bacilli were detected as part of a case with disseminated disease, although the exact neurological signs were not described.^12,20^ No organisms were identified within the neural tissues examined in another case, which was reported to have hindlimb ataxia and spinal pain.^5^ In the current case, while there were no granulomatous lesions within the CNS, the presence of mycobacteria in some of the neural tissues examined supports MAC infection as the cause of the cat’s neurological signs. Central nervous system involvement is therefore a potential, albeit rare, manifestation of MAC infection, which has also been seen in another cat by one of the authors (DGM; unpublished results); it is believed to be caused by haematogenous spread of the bacteria. Neurological signs will therefore be highly variable and dependent on location.

Infections with MAC in different species (including humans and cats) are strongly associated with profound immunosuppression of the host; however, no association has been found between feline MAC cases and FIV/FeLV status.^21,22^ While the cat in this study was negative for FIV and FeLV on in-house testing, it was receiving long-term immunosuppressive therapy with ciclosporin. One previous case report also featured a cat receiving long-term ciclosporin therapy; it had a rapidly progressive clinical presentation and was euthanased shortly after presentation.^7^ It was theorised that ciclosporin treatment altered the cat’s innate immunity by indirect inhibition of the activation and proliferation of T cells; this is important as cats are normally naturally resistant to infection with *M avium*.^23^

The decision to use the combination of a fluoroquinolone and azithromycin pending speciation of the mycobacteria was based on current recommendations for the treatment of feline mycobacterial diseases.^22,24^ While rifampin therapy was considered, given the potential side effects, treatment was withheld until the species of mycobacteria was known.

In humans, clarithromycin is the antibiotic of choice for treating disseminated MAC infections and, as with other mycobacterial infections, double or triple antibiotic combinations are used for synergistic effects.^24,25^ Traditionally, fluoroquinolones form part of the treatment for MTBC pathogens; however, they have been found to be ineffective against MAC.^26^ Various antibiotic regimens have been used in the treatment of disseminated *M avium* infection in cats and, while treatment failure is a frequent occurrence, successful outcomes have been reported.^4,18^ Single drug therapy is not advised, and the current treatment recommendation for feline MAC is a multidrug protocol over a prolonged period of at least 6 months.^21^ As in humans, clarithromycin is the cornerstone of treatment in cats, and clinical success has been documented using clarithromycin in double or triple combinations with clofazimine, rifampin or doxycycline; the newer fluoroquinolones (eg, pradofloxacin in cats and moxifloxacin in humans) have also been suggested as treatment options.^2,4,18^

The rapid clinical progression in this case was likely due to the delay in investigation and the disseminated nature of the infection at the time of referral, rather than representing a treatment failure per se. The decision to continue with ciclosporin therapy was undoubtedly a factor in the rapid clinical progression of infection.

While MAC can cause infection in a wide range of different animal species, including humans, the zoonotic potential of pets is unclear.^21^ There is no evidence to suggest that transmission has ever occurred from an infected cat to a human and, likewise, infection from cat to cat is also considered unlikely.^2,21^ Suggested sources of infection in companion animals are from the ingestion of infected meat, prey species or contact with infected soil or fomites. MAC can remain viable in the environment for at least 2 years.^21^ Given their shared environment, a common source of infection is considered very likely in these cats.

A study looking at the geographical distribution of feline mycobacterial infections in the UK reported a large cluster of *M avium* cases in eastern England; 66% of the isolates in this area were *M avium* vs an overall UK prevalence of 15%.^3^ While the cats in the current report had been living in Scotland for 3 years at the time of diagnosis, they had previously lived within this eastern England zone for approximately 5 years. Their only known hunting history was access to a bird nest when they lived in eastern England, so they may have had direct contact with birds and their fomites at that point.

Mycobacteria can sometimes lead to infections that lay dormant for many years, before clinical disease becomes apparent. It is well recognised in humans that *M tuberculosis* can cause a latent infection that can activate, or reactivate in some cases, although this mechanism is poorly understood.^27^ Likewise, MAC infections in humans may also have a latent period.^28^ Latent (or inactive) infections and late presenting infections have also been seen in cats with *M bovis* infection.^29,30^ It is therefore reasonable to assume that latency, with the potential to reactivate, could also occur with MAC infections in cats. It is tempting to speculate that both cats were exposed to the mycobacteria from a common source when they lived in eastern England and both developed latency, with the infection activating several years later in the female cat as a result of long-term immunosuppressive therapy.

A latent MAC infection was suspected in the second cat based on the IGRA result in the absence of any detectable disease. This is similar to two cats in another report where the positive IGRA was suspicious for exposure only.^31^ This IGRA has been shown to have a sensitivity and specificity of 66.7% and 92.6%, respectively, for detecting NTM infections (updated to 83.3% and 93.3%, respectively, after cut-off adjustments were made in a recent study); however, it cannot differentiate between different NTM species (ie, it cannot specifically detect *M avium* or even MAC).^31^ However, as *M avium* was confirmed by PCR in the other cat, and as both cats had only one known exposure, the likelihood of the second cat having been exposed to a different NTM is unlikely. The sensitivity and specificity of the IGRA to differentiate the NTM are lower than those reported for MTBC infections; because MAC is a large complex of related infections, it may be this marked heterogeneity, compared with the marked homogeneity of the TB complex, that makes the IGRA less sensitive and specific for this complex.^31^

There are currently no guidelines available for situations where an animal is IGRA test positive for MAC but has no visible disease. Options suggested for cats that are IGRA positive for *M bovis* include close monitoring only, prophylactic treatment with isoniazid (which is sometimes used in humans for this purpose) or appropriate antibiotic therapy for 3 months; however, there is no evidence on which to base this decision.^32^ The same principles could also be applied to scenarios with MAC-positive IGRA cats, although appropriate antibiotic therapy is likely to differ between *M bovis* and MAC cases. Prophylactic treatment with isoniazid may have little effect against latent MAC infections. In humans, isoniazid is suggested for prophylactic tuberculosis treatment, but has no place in the treatment of MAC, whereas rifabutin is recommended instead (eg, for the prophylactic treatment for MAC infections in people with HIV).^33,34^ It may therefore be a more appropriate antibiotic choice.The decision to simply monitor the in-contact cat in this report was agreed between the authors and the owner.

## Conclusions

This case highlights the need to include disseminated MAC infection as a differential diagnosis for weight loss and (peripheral or internal) lymphadenopathy in cats, particularly if receiving immunosuppressive therapy such as ciclosporin. Late presentations of disease should also be considered, and so detailed histories should be taken by clinicians.

CNS involvement is a rare complication of disseminated MAC infection, and clinicians should be aware of the wide range of clinical findings that can be associated with disseminated MAC infections such as those described herein.

In-contact cats should be screened for infection as latent infections are possible and the options for managing these cases should be carefully discussed with the owners.
